# Biological and Computational Studies for Dual Cholinesterases Inhibitory Effect of Zerumbone

**DOI:** 10.3390/nu12051215

**Published:** 2020-04-25

**Authors:** Jayeong Hwang, Kumju Youn, Yeongseon Ji, Seonah Lee, Gyutae Lim, Jinhyuk Lee, Chi-Tang Ho, Sun-Hee Leem, Mira Jun

**Affiliations:** 1 Department of Food Science and Nutrition, Dong-A University, Busan 49315, Korea; 2 Center for Silver-Targeted Biomaterials, Brain Busan 21 Plus Program, Graduate School, Dong-A University, Busan 49315, Korea; 3 Korean Bioinformation Center, Korea Research Institute of Bioscience and Biotechnology (KRIBB), Daejeon 34141, Korea; 4 Department of Bioinformatics, KRIBB School of Bioscience, Korea University of Sciences and Technology, Daejeon 34113, Korea; 5 Department of Food Science, Rutgers University, New Brunswick, NJ 08901, USA; 6 Department of Biological Science, Dong-A University, Busan 49315, Korea

**Keywords:** ADMET, Alzheimer’s disease (AD), cholinesterases, computational docking simulation, zerumbone

## Abstract

Acetylcholinesterase (AChE) and butyrylcholinesterase (BChE) mediate the degradation of acetylcholine (ACh), a primary neurotransmitter in the brain. Cholinergic deficiency occurs during the progression of Alzheimer’s disease (AD), resulting in widespread cognitive dysfunction and decline. We evaluated the potential effect of a natural cholinesterase inhibitor, zerumbone, using in vitro target enzyme assays, as well as in silico docking and ADMET (absorption, distribution, metabolism, excretion, and toxicity) simulation. Zerumbone showed a predominant cholinesterase inhibitory property with IC_50_ values of 2.74 ± 0.48 µM and 4.12 ± 0.42 µM for AChE and BChE, respectively; however, the modes of inhibition were different. Computational docking simulation indicated that Van der Waals interactions between zerumbone and both the cholinesterases were the main forces responsible for its inhibitory effects. Furthermore, zerumbone showed the best physicochemical properties for both bioavailability and blood–brain barrier (BBB) permeability. Together, in the present study, zerumbone was clearly identified as a unique dual AChE and BChE inhibitor with high permeability across the BBB, suggesting a strong potential for its physiological benefits and/or pharmacological efficacy in the prevention of AD.

## 1. Introduction

Alzheimer’s disease (AD) is a fatal neurodegenerative disease characterized by an irreversible degeneration of neurons, leading to the impairment of cognitive functions. Major pathological features of AD include the extracellular accumulation of amyloid-β peptide (Aβ) in senile plaques and the intracellular deposition of hyperphosphorylated tau protein in neurofibrillary tangles. Specifically, amyloid precursor protein (APP) proteolysis into Aβ by β- and γ-secretases, forms the initiating pathological event in AD [1]. 

Acetylcholine (ACh), the most abundant primary neurotransmitter in the brain, is responsible for cholinergic transmission. Cholinesterase enzymes, including acetylcholinesterase (AChE) and butyrylcholinesterase (BChE), catalyze the hydrolysis of Ach [2]. In the normal human brain, AChE has a 10-fold higher ACh hydrolytic activity compared to that of BChE; however, the activity of the former decreases and the latter increases, to compensate for the AChE activity in cholinergic neurons during AD development [3]. However, abnormally high activity of AChE has been observed around amyloid plaques in AD patients [4]. Furthermore, AChE has promoted Aβ aggregation and produced stable AChE-Aβ complexes that are more neurotoxic than Aβ itself [5]. 

The blood–brain barrier (BBB) modulates CNS homeostasis by severely controlling the transport of compounds to the brain [6]. Limited brain exposure, toxicity, as well as low bioavailability form the major factors that restrict the in vivo efficacy of anti-AD agents at the target sites. Conventional approaches involving the biological assessment of numerous compounds have proven to be time-consuming and cost ineffective, with a low probability of success. Thus, prior evaluation of the physicochemical characteristics of candidate compounds, as well as the analysis of their pharmacokinetic and toxicity properties, is a crucial prerequisite for the screening and selection of potential anti-AD agents 

Zerumbone is a sesquiterpene, abundantly found in the essential oil of the wild ginger, *Zingiber zerumbet* (L) Smith. The compound has been found to exert a broad range of potential biological effects, such as hepatoprotective, antioxidant, immunoregulatory, anti-inflammatory, and anticancer activities [7,8,9,10]. Despite numerous studies on zerumbone, its direct effect on cognitive functions and dementia has not yet been elucidated. Therefore, the present study focused on evaluating the inhibitory effect of zerumbone on AChE and BChE activities. In addition, the ability of zerumbone to reach the target site and interact with the targeted enzymes was investigated using in silico docking and ADMET simulation tools.

## 2. Materials and Methods

### 2.1. Reagent

Zerumbone (≥98% purity) and galantamine were purchased from Sigma-Aldrich (St. Louis, MO, USA). The target enzymes AChE, BChE, and their substrates, as well as elastase, trypsin, chymotrypsin, and their substrates were also obtained from Sigma-Aldrich (St. Louis, MO, USA). A BACE1 assay kit was obtained from Thermo Fisher Scientific, Inc. (Waltham, MA, USA).

### 2.2. Enzyme Kinetics and Substrate Selectivity

An anti-cholinesterase activity assay was modified from an Ellman method [11]. AChE or BChE (0.8 U/mL) was added to a mixture which contained 0.1 M sodium phosphate buffer, 5 mM DTNB, and a tested sample, and then incubated at 37 °C for 15 min. AChE iodide or BChE iodide (750 μM) was then added to the mixture to activate the reaction. The enzymatic hydrolysis of AChE iodide or BChE iodide was observed by tracking the production of a colored anion (5-thio-2-nitrobenzoic acid) that resulted from the reaction between DTNB and thioiodide, which was released by the enzyme, using a microplate spectrophotometer (ELX808, Winooski, VT, USA) at 405 nm.

To evaluate BACE1 inhibitory activity, a BACE1 FRET assay kit with a recombinant baculovirus-expressed BACE1 and a Rh-EVNLDAEFK-Quencher substrate was used [12]. Briefly, a mixture of BACE1 (1.0 U/mL), the substrate, and tested sample at a different concentration, was incubated for 60 min at 25 °C in well plates. The increase in fluorescence intensity was measured by a fluorescence microplate reader at excitation and emission wavelengths of 545/590 nm (FLX800, Winooski, VT, USA). The inhibitory ratio (%) was calculated using the formula: Inhibition (%) = [1 − (S − S_0_)/(C − C_0_)] × 100, where C is the absorbance of control after 60 min of incubation, C_0_ is the absorbance of control at time 0, S was the absorbance of samples after 60 min of incubation, and S_0_ was the absorbance of the samples at time 0. 

To determine enzyme selectivity, trypsin, chymotrypsin, and elastase were analyzed using N-benzoyl-L-Tyr-pNA, N-benzoyl-L-Arg-pNA, and N-succinyl-Ala-Ala-Ala-pNA as substrates, respectively. The reaction mixture was incubated for 20 min and the absorbance was measured at 410 nm [12].

The cholinesterases inhibition kinetics of zerumbone were analyzed using Dixon and Lineweaver-Burk plots. The dissociation constant (Ki) was determined by interpretation of the Dixon plot, and Vmax and Km were calculated by Lineweaver–Burk plots, using the initial velocities obtained over a substrate concentration range [11]. These kinetic parameters were calculated using the SigmaPlot™ version 12.3 (Systat Software, Inc., San Jose, CA, USA). 

### 2.3. ADMET Predictions and Molecular Docking Study

The pharmacokinetic properties including absorption, distribution, metabolism, excretion, and toxicity (ADMET) of zerumbone were calculated by pkCSM (http://biosig.unimelb.edu.au/pkcsm/). pkCSM is a novel method for predicting and optimizing small molecule ADMET properties which rely on distance-based graph signatures. The pkCSM tool adopted the concept of cutoff scanning to indicate 30 predictors, which were classified into five main categories: absorption (7 parameters), distribution (4 parameters), metabolism (7 parameters), excretion (2 parameters), and toxicity (10 parameters) [13].

The Autodock Vina program (version 1.1.2) was used to predict the bound conformation and the lowest binding energy of the target enzyme–ligand complex. Pck software was conducted to search the binding pocket residues of enzymes. The chemical structure of zerumbone was drawn, displayed, and characterized using Marvin Sketch (5.11.4, 2012, ChemAxon, One Broadway Cambridge, MA, USA). The grid box of dimensions was set to x = 30 Å, y = 30 Å, and z = 30 Å. The Protein Data Bank (PDB) ID of 3D structures of human AChE and BChE were 4PQE and 1P0I, respectively. The crystal structure of zerumbone was gained from PubChem (CID 5470187) [14].

### 2.4. Statistics

All data were represented as the mean ± standard deviation (SD) in three independent experiments, each performed in triplicates. Statistical analyses were determined using Duncan’s multiple range tests using the Statistical Analysis System (SAS), version 9.3 (Cary, NC, USA).

## 3. Results and Discussion

### 3.1. In Vitro Cholinesterase Inhibitory Property of Zerumbone

**Figure 1:**
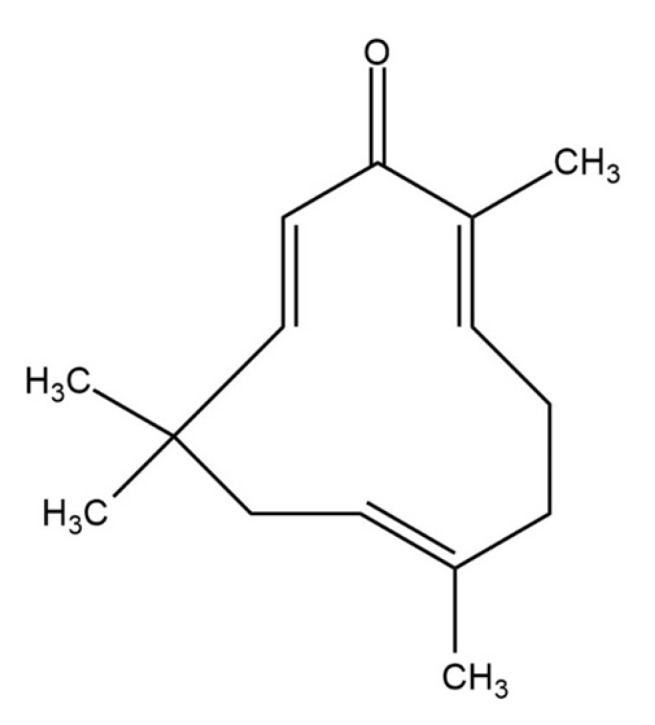
The chemical structure of zerumbone.

Zerumbone is a sesquiterpene compound found in the essential oils of *Z. zerumbet* in the range of 35.5% to 84.8%. The chemical structure of zerumbone is represented in Figure 1. The novel finding in our study was that zerumbone plays as a unique dual AChE and BChE inhibitor with strong IC_50_ values (2.74 ± 0.48 µM and 4.13 ± 0.42 µM, respectively) (Table 1). The compound exhibited an approximately 3-fold more potent BChE suppression in comparison to that of the positive control, galantamine (IC_50_ 14.47 ± 0.33 µM). In considering the wide IC_50_ ranges of published natural AChE inhibitors as positive controls, including flavonoids, coumarins, alkaloids, terpenoids, and polyphenols (from 0.28 µM to >200 µM); zerumbone was identified as a potent AChE inhibitor. The unique chemical structure comprising of an α, β-unsaturated carbonyl group at the C-8 position may account for the high inhibitory potency of zerumbone against cholinesterases. α-Humulene, a structural analog lacking only the concerned carbonyl group in zerumbone, was virtually inactive in oxidative stress related inflammation and cancer cell proliferation, suggesting that the α, β-unsaturated carbonyl group in zerumbone might have a crucial role in interacting with the targets [15].

To assess the enzyme specificity of zerumbone, its inhibitory activities on trypsin, chymotrypsin, elastase, and β-secretase 1 (BACE1) were evaluated. Zerumbone did not exhibit any inhibitory activity on the above-mentioned enzymes, suggesting that the compound is selective and specific against AChE and BChE (Table 2).

### 3.2. Enzyme Inhibition Kinetics of Zerumbone

**Figure 2:**
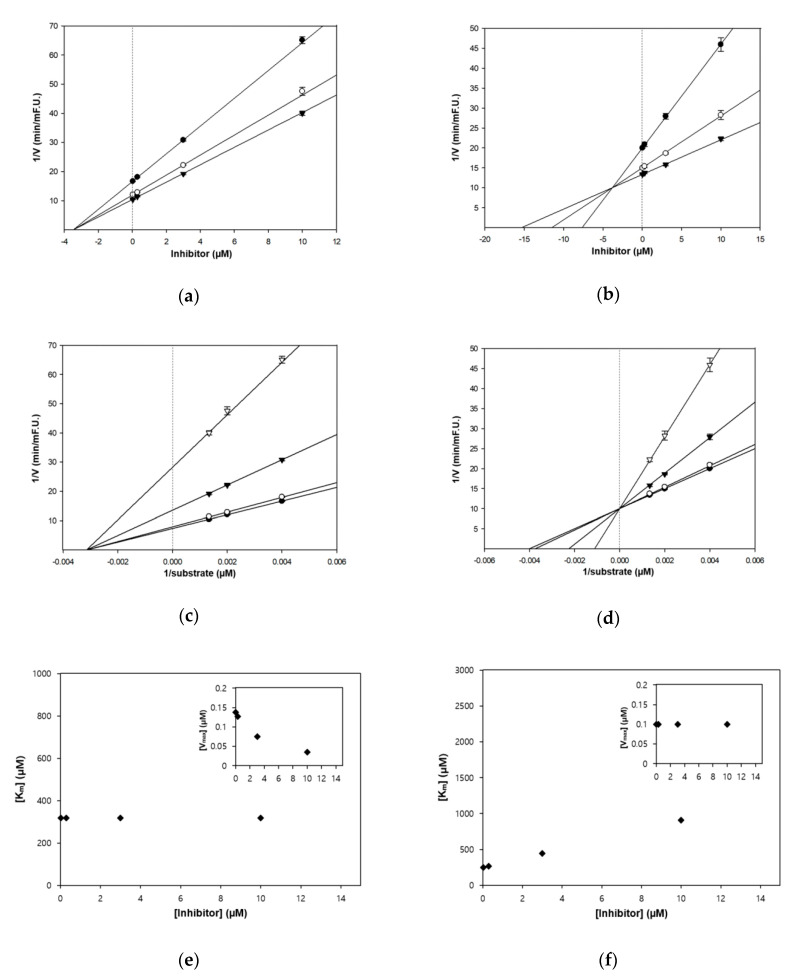
The kinetics of AChE and BChE inhibition by zerumbone. Dixon plots represent the effects of increasing substrate concentrations: 250 µM (●); 500 µM (○); and 750 µM (▼) for zerumbone (**a**,**b**). Lineweaver–Burk plots were evaluated in the presence of the inhibitor at different concentrations: 0.03 μM (●); 0.3 μM (○); 3 μM (▼); and 10 μM (▽) for zerumbone (**c**,**d**). The Km values, and the dependence of the values of Vmax, on the concentration of zerumbone ((Inset) (**e**,**f**)).

As shown in Table 1, the inhibition type of zerumbone was assessed using kinetics experiments. The three lines of Dixon plot presented the same x-intercept with a Ki value of 3.5 μM (Figure 2). In addition, the Lineweaver-Burk plot and its secondary replot indicate that zerumbone is a non-competitive inhibitor of AChE, thereby interacting at sites other than the active binding sites of AChE. On the other hand, zerumbone exhibited a competitive type inhibition for BChE with a Ki value of 3.8 μM, implying that our targeted compound is structurally related to the substrate and competes for occupying the active site of BChE [16].

### 3.3. Molecular Docking Studies for Zerumbone

**Figure 3:**
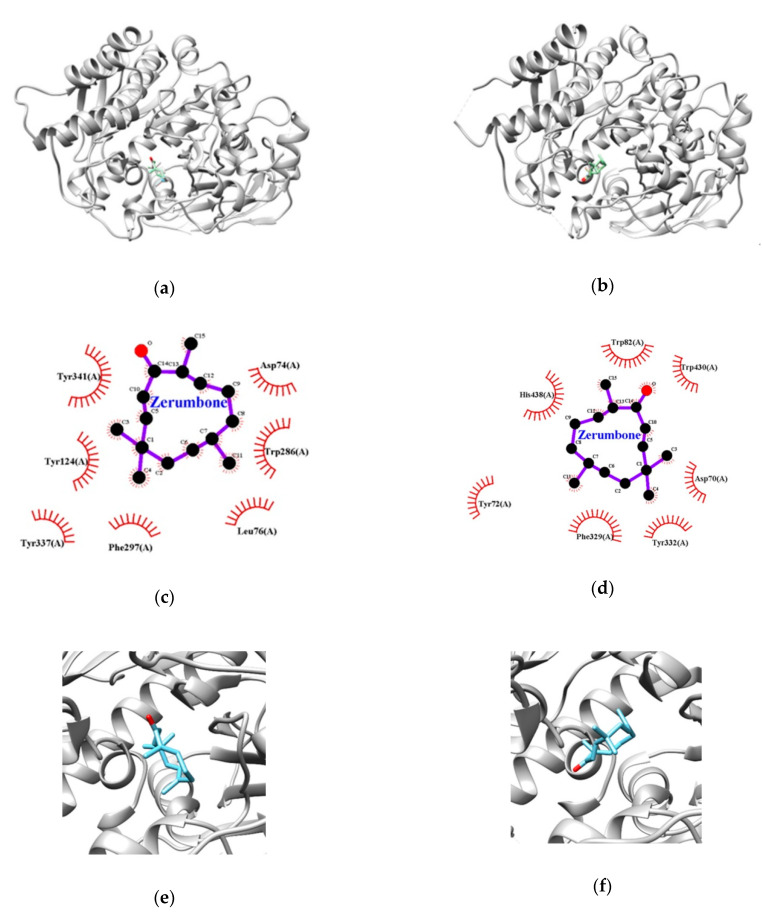
Representations of the best poses of zerumbone in docking with AChE (**a**) and BChE (**b**). Zoomed in view of the zerumbone binding site at AChE (**c**) and BChE (**d**). The hydrophobic interaction between AChE (**e**), BChE (**f**), and zerumbone. The hydrophobic interactions are depicted as red dashed semicircles.

Computational docking analysis of zerumbone with the target enzymes predicted the conformation that best fits the binding sites and estimated the stability of the ligand−enzyme complexes. To better understand the biological activity of zerumbone against AChE and BChE, a docking simulation was conducted. The lowest binding energies of zerumbone were −8.0 kcal/mol with AChE and −7.6 kcal/mol with BChE, suggesting a high affinity for both enzymes (Table 3 and Figure 3). In the zerumbone−AChE complex, eight residues of AChE including TYR72, ASP74, LEU76, TYR124, TRP286, PHE297, TYR337, and TYR341 were demonstrated to participate in Van der Waals interactions with the compound.

AChE contains two substrate binding sites, namely, the catalytically active site (CAS) that is responsible for neurotransmitter degradation, and the peripheral anionic site (PAS) that is responsible for the non-cholinergic functions of AChE. The PAS binding site stimulates the Aβ aggregation and senile plaques formation, and thus increases the toxicity of Aβ. The present molecular docking results demonstrated that zerumbone occupies the PAS region of AChE, including TYR72, ASP74, TYR124, TRP286 and TYR341, which is consistent with the in vitro experimental results, indicating zerumbone to be a non-competitive inhibitor. 

Moreover, the zerumbone−BChE complex was found to be stabilized by the formation of Van der Waals interactions with ASP70, TRP82, PHE329, TYR332, TRP430, and HIS438. The important active site residues of BChE were divided into three groups, including catalytic triads (SER198, HIS438 and GLU325), acyl binding pockets (GLY116, GLY117, TRP231, LEU286, and VAL288), and a choline binding pocket (TRP82). Interestingly, zerumbone bound closely with one of the catalytic triads (HIS438) and the choline binding pocket (TRP82) of BChE, implying that the mode of action is competitive.

### 3.4. In Silico ADMET Predictions for the Bioavailability of Zerumbone

The designing of novel anti-AD candidates requires substantial attention to their pharmacokinetic properties. ADMET parameters were evaluated to assess the bioavailability of zerumbone. As shown in Table 4, zerumbone was predicted to possess good water solubility (−4.03 log mol/L), with appreciable absorption from the intestine (Caco-2 permeability > 0.90 × 10^−6^ cm/s and human intestinal absorption >30%). The skin permeability value (−2.06 log Kp) of zerumbone, which defines the rate of a chemical passage through the stratum corneum, was comparable with that of the standard value (−2.5 log Kp), showing its potential as a lead structure and justifying its drug-likeliness behavior [17].

In distribution properties, volume of distribution (VDss) was considered. VDss represents the total volume required by the drug concentration to be uniformly distributed to give the same concentration at the target site as in the plasma [18]. Higher values of VDss indicate that most of the drug is contained in tissues rather than in plasma. For zerumbone, the unbound fraction in plasma and the steady state volume of distribution (log VDss < 0.28) was low enough to have a high plasma protein affinity. 

The BBB and CNS permeability values were also calculated for zerumbone and compared with the respective standard values. It has been reported that candidates with a >0.3 log BB value have the potential to pass the BBB, while those with a less than −1 value are poorly distributed to the brain. In addition, candidates that have > −2 log PS value are considered to have penetrated the CNS, while those with < −3 value have difficulty in penetrating the CNS. Fortunately, zerumbone was observed to be blood−brain positive (0.52 log BB) and CNS permeable (−2.65 log PS). 

The non-mutagenic and non-toxic behavior of zerumbone was confirmed from the predicted Ames toxicity. Zerumbone did not act as a substrate or inhibitor for the hepatic cytochrome P450 isoenzymes. Further, no hepatotoxicity characterized by disrupted normal liver functions was observed. The predicted median lethal concentration (LC_50_) of zerumbone did not fall within the range of toxicity. The potential relevance of zerumbone was observed in several previous in vivo studies which demonstrate the administration of the compound did not produce significant acute toxicity [19,20,21]. Zerumbone did not cause any genotoxicity, nor did it cause significant changes in organ weight, clinical chemistry, hematology, or histopathological observations, after single or repeated intraperitoneal injection in mice. 

Promising candidates are too often withdrawn after their market launch for a variety of reasons, including low bioavailability, high toxicity, poor pharmacokinetics, or drug–drug interactions [21,22]. For example, the well-known AChE inhibitors, physostigmine and tacrine, were withdrawn from the market due to poor bioavailability and hepatotoxicity [23]. In addition, the advent of parallel synthesis techniques and high throughput screening has placed increasing stress on the method that has traditionally been used to evaluate potential candidates at the pre-clinical stage [24]. Because of the limited time and increased costs available to perform formal in vivo studies, generally tens of candidates will be selected. Therefore, computational prioritization, prior to biological evaluation, is important in order to confirm that resources are apportioned to the most promising candidates [25]. Moreover, while the binding properties of candidates for the therapeutic target are important, ensuring that they can reach the target site in sufficient concentrations to provide the therapeutic effect safely, is crucial for their introduction into the clinical trial [26]. Therefore, appreciation of the importance of ADMET properties in early stage drug development has led to a significant reduction in the number of compounds that have failed in clinical trials due to poor pharmacokinetics [13].

Recent study has suggested that administration of zerumbone (1 and 10 mg/kg) attenuated cognitive impairments and memory deficits related to scopolamine, an anticholinergic agent, in Sprague–Dawley rats [27]. Another study showed that the compound (25 mg/kg) alleviated behavioral impairments and Aβ deposition by inhibiting the MAPK signaling pathway in transgenic APP/PS1 mice [28]. 

The multifactorial etiology of AD indicates that a multi-targeted approach might be more advantageous than a single-targeted approach. The current study is the first investigation to demonstrate the potential anti-AD efficacy of zerumbone as a strong dual inhibitor of both AChE and BChE with different modes of action. Moreover, zerumbone exhibited optimal pharmacokinetic profiles as well as relatively low toxicity in silico, suggesting that the compound could be considered as a lead compound for the prevention of AD.

## 5. Conclusions

AChE and BChE form one of the therapeutic targets for the management of cognitive dysfunctions in AD. Due to the unique dual enzyme inhibition conferred by zerumbone, the compound has distinctive advantages over the previously reported single enzyme-targeted inhibitors. Furthermore, our positive results of ADMET prediction for zerumbone support its potential implication as a preventative agent in AD.

## Figures and Tables

**Table 1:** Cholinesterases inhibitory activities, inhibition type, and dissociation constants (Ki) of zerumbone.

Sample	AChE			BChE		
	IC_50_^1^	K_i_ Value ^2^	Inhibition Type ^3^	IC_50_	K_i_ Value	Inhibition Type
Zerumbone	2.78 ± 0.48	3.5	Non-competitive	4.12 ± 0.42	3.8	Competitive
Galantamine **^4^**	1.50 ± 0.05	-	Competitive	14.47 ± 0.33		Competitive ^5^

^1^ IC_50_ (µM) was indicated as a mean ± standard deviation (SD) of the independent triplicate experiments. ^2^ The inhibition constants (Ki, µM) represented the binding affinity between the inhibitor and the enzyme. ^3^ The inhibition type was obtained by Dixon and Lineweaver-Burk plots. ^4^ Galantamine was used as a positive control in the cholinesterase assays. ^5^ Ref. Synthesis and Evaluation of the Biological Profile of Novel Analogues of Nucleosides and of Potential Mimetics of Sugar Phosphates and Nucleotides. 2015. Xavier et al. (-) Not tested.

**Table 2:** The inhibitory activity (%) ^1^ of zerumbone against serine proteases and BACE1.

Compounds (µM)		Trypsin	Chymotrypsin	Elastase	BACE1
Zerumbone	50	5.00 ± 1.94	3.45 ± 0.30	3.46 ± 1.85	13.35 ± 1.20
	100	3.75 ± 2.03	2.71 ± 0.45	3.57 ± 0.94	16.08 ± 1.36

^1^ The inhibitory activity (%) is expressed as mean ± SD.

**Table 3:** The molecular interaction of cholinesterases with zerumbone.

Enzymes	Lowest Energy (Kcal/mol)	Van der Waals Residues
AChE	−8.0	TYR72, ASP74, LEU76, TYR124, TRP286, PHE297, TYR337, TYR341
BChE	−7.6	ASP70, TRP82, PHE329, TYR332, TRP430, HIS438

**Table 4:** In silico ADMET properties of zerumbone.

	Properties	Predicted Values
Absorption	Water solubility	−4.03 (log mol/L)
	Permeability of Caco-2	1.43 (log Papp in 10^−^^6^ cm/s)
	Human intestinal absorption	95.78 (% Absorbed)
	Skin permeability	−2.06 (log Kp)
	Substrate of P-glycoprotein (P-gp)	No
	Inhibitor of P-gp I, II	No
Distribution	Volume of distribution (VDss, human)	0.28 (log L/kg)
	Fraction unbound (human)	0.40 (Fu)
	Permeability of BBB	0.52 (log BB)
	CNS permeability	−2.65 (log PS)
Metabolism	Substrate of CYP2D6/CYP3A4	No
	Inhibitor of CYP1A2/CYP2C19/CYP2C9/CYP2D6/CYP3A4	No
Excretion	Total clearance	1.31 (log ml/min/kg)
	Renal OCT2 substrate ^1^	No
Toxicity	Ames toxicity ^2^	No
	Maximum tolerated dose (human)	0.53 (log mg/kg/day)
	Inhibitor of hERG ^3^ I/II	No
	Hepatotoxicity	No
	Oral Rat Acute Toxicity (LD_50_)	1.08 (mol/kg)
	Oral Rat Chronic Toxicity (LOAEL)	1.18 (log mg/kg_body weigh)t/day)
	Skin Sensitization	Yes
	*T. Pyriformis* toxicity (IGC_50_) ^4^	1.39 (log µg/L)
	Minnow toxicity (LC_50_) ^5^	1.03 (log mM)

^1^ OCT is organic cation transporter. ^2^ A compound which tests positive for the Ames mutagenicity test is mutagenic and therefore may act as a carcinogen. ^3^ A hERG (human Ether-à-go-go-Related Gene) I/II inhibitor could cause the development of the acquired long QT syndrome, which leads to fatal ventricular arrhythmia. ^4^ *T. Pyriformis* (*Tetrahymena Pyriformis*) is a protozoa bacterium. ^5^ LC_50_, the median lethal concentrations, indicates the concentration of a substance necessary to cause 50% mortality of flathead minnows.
